# Computational modeling of human reasoning processes for interpretable visual knowledge: a case study with radiographers

**DOI:** 10.1038/s41598-020-77550-9

**Published:** 2020-12-10

**Authors:** Yu Li, Hongfei Cao, Carla M. Allen, Xin Wang, Sanda Erdelez, Chi-Ren Shyu

**Affiliations:** 1 Department of Electrical Engineering and Computer Science, University of Missouri, Columbia MO, 65211 USA; 2 Institute for Data Science and Informatics, University of Missouri, Columbia MO, 65211 USA; 3 Department of Clinical and Diagnostic Science, University of Missouri, Columbia MO, 65211 USA; 4 Department of Information Science, University of Northern Texas, Denton TX, 76203 USA; 5 School of Library and Information Science, Simmons University, Boston, MA 02115 USA

**Keywords:** Computational science, Computer science

## Abstract

Visual reasoning is critical in many complex visual tasks in medicine such as radiology or pathology. It is challenging to explicitly explain reasoning processes due to the dynamic nature of real-time human cognition. A deeper understanding of such reasoning processes is necessary for improving diagnostic accuracy and computational tools. Most computational analysis methods for visual attention utilize black-box algorithms which lack explainability and are therefore limited in understanding the visual reasoning processes. In this paper, we propose a computational method to quantify and dissect visual reasoning. The method characterizes spatial and temporal features and identifies common and contrast visual reasoning patterns to extract significant gaze activities. The visual reasoning patterns are explainable and can be compared among different groups to discover strategy differences. Experiments with radiographers of varied levels of expertise on 10 levels of visual tasks were conducted. Our empirical observations show that the method can capture the temporal and spatial features of human visual attention and distinguish expertise level. The extracted patterns are further examined and interpreted to showcase key differences between expertise levels in the visual reasoning processes. By revealing task-related reasoning processes, this method demonstrates potential for explaining human visual understanding.

## Introduction

Human visual processing is critical in the reasoning and decision making for many tasks and attracts researchers from various disciplines. Studies in psychology and neural science show that visual attention is heavily affected by both visual features and visual tasks^1^, as well as accumulated experience^2^. In computer science, people have been able to model and predict human visual attention regarding different levels of visual features^3^, emotion^4,5^, and viewing time^6^. However, many studies target simple visual tasks which cannot fully reveal the reasoning processes behind complex visual tasks.

One of the areas needing such understanding is medical image interpretation. Medical imaging specialists accumulate implicit knowledge of highly complicated visual tasks through years of experience with real cases, which makes them irreplaceable despite recent developments of computer vision and clinical decision support systems which heavily rely on black-box algorithms^7,8^. Lack of explainability is one major concern preventing these systems from being widely adopted in critical areas^9^, especially where the human reasoning processes are not well standardized such as radiology and pathology. For such complex tasks, the visual reasoning processes may differ depending on the task and level of expertise. In some cases, there can be diagnostic discrepancies among different experts or even between two diagnoses by the same expert^10^. One major source for error (around 60%) in radiology is the radiologists’ perception^11,12^. The main method for identifying the causes of the errors is case-by-case analysis in which experts need to closely examine and discuss the images with discrepancies^13–15^. There is an urgent need to understand and formalize the visual reasoning processes behind medical image interpretation, and an efficient computational method is essential to provide insight and evidence for this purpose^16^.

As an unobtrusive method that can be seamlessly integrated into natural workflow, eye movement analysis has been applied to medical diagnosis processes to investigate visual decision making^17–19^, model perceptual behavior^20,21^, and improve medical interpretation^22,23^. Previous studies show the applicability of eye movement in decoding visual attention, but some are limited by their methods which either require extensive analysis of entire eye movement sequences or lack quantified measurements to capture the temporal or spatial differences^24^. To this point, many studies have been undertaken to extract detailed reasoning processes, but the methods and metrics employed could only support identification of general visual behaviors supported by saliency maps or time consumption for fixations and saccades^25^.

There have been a number of eye movement analysis methods employed in the field of eye tracking studies^26–34^. Many methods focus on clustering similar eye movement sequences^26–28,30^, and some can produce explainable results that can be understood by medical specialists^31–34^. The extracted eye movement sequences from some of the methods with explainable results could be a high-level explanation abstracted from multiple sequences^31,32,35^. Given that some sequences could contain prolonged scanning and search periods and may not always focus on task-related regions, such results can potentially omit important details in subsequences. We identify a need for a new computational method to produce task-related, explainable visual reasoning patterns.

We propose a visual knowledge and reasoning discovery system to efficiently identify cases with significant differences and extract common and unique visual reasoning patterns. We adapt the Markov Chain (MC) and graph models to quantify the spatial and temporal differences of eye movement sequences. This provides a quantitative measure on how people differ in certain tasks and a reference for locating significant cases. The system also extracts common and unique patterns of eye movement exhibited frequently by all viewers or only in certain viewer groups. The extraction process filters out irrelevant or transitional fixations while focusing only on the prevalent eye movements which fulfil certain goals. The extracted patterns are original eye movement sequences which are explainable and can be translated into visual strategies. These patterns can support understanding the visual reasoning processes and provide data for evidence-based medical image interpretation. The method is a first step toward our effort to design computational methods with explainable reasoning processes.

The rest of the paper is structured as follows. In Method, we introduce the system architecture and explain the components of data preprocessing, visual reasoning quantification, and pattern extraction. The Results section shows the effectiveness of quantification in differentiating visual reasoning processes and explains the significant patterns with expertise progression. In Discussion, the results are summarized, and the prospects of the methods are discussed.

## Method

**Figure 1:**
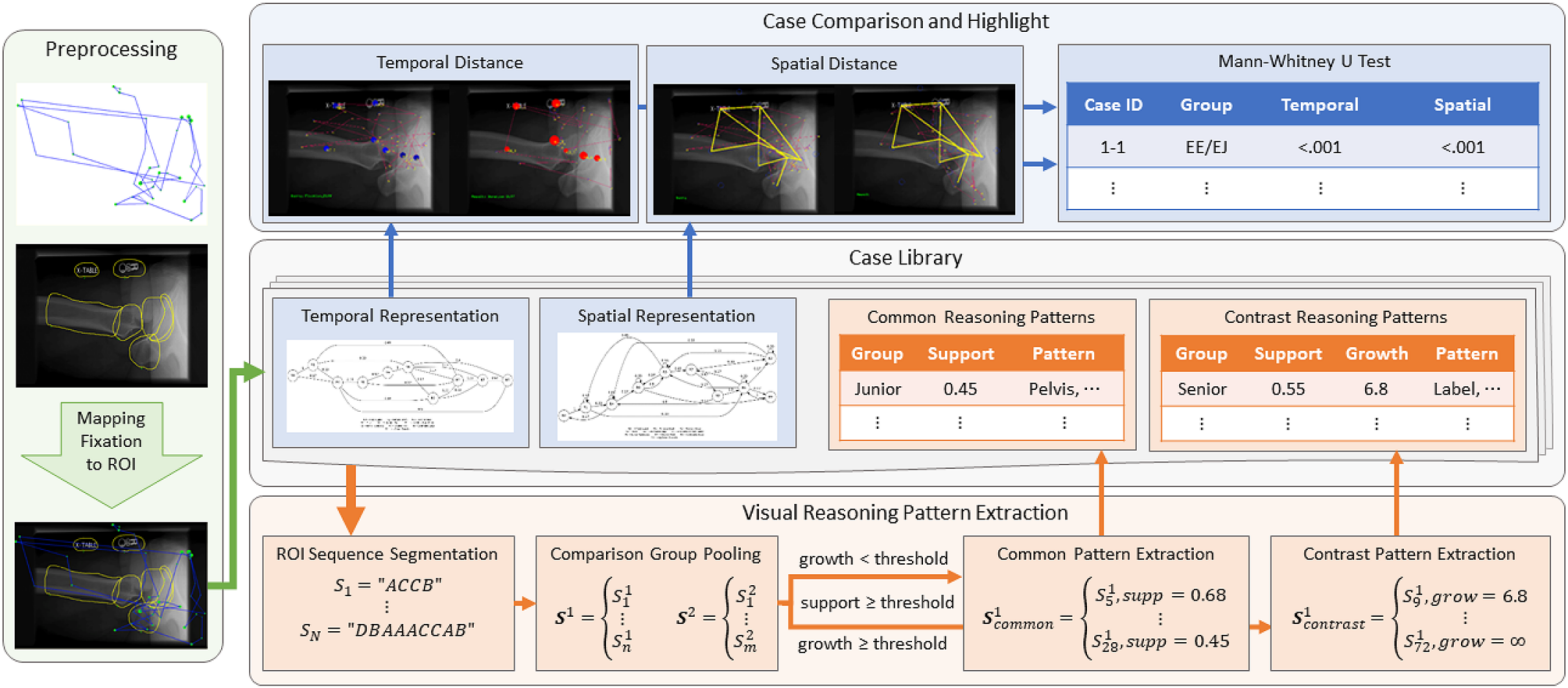
The system architecture of visual reasoning quantification and extraction.

The overall architecture of our proposed visual reasoning comparison and extraction system is shown in Fig. 1, which includes four components: raw data preprocessing, case library, visual reasoning comparison, and reasoning pattern extraction. Each case consists of a problem description, domain image and gaze tracking data. After processing, the temporal and spatial representation, as well as extracted patterns are attached to the case.

The system adapts the methods in our previous work by Cao et al.^36^ to quantify the spatial and temporal characters of the visual reasoning cases. To measure the spatial distance by a graph representation, we adapted a subgraph distance: Substructure Index-based Approximate Graph Alignment (SAGA)^37^. Three components were designed to measure the differences of properties from two visual reasoning processes. For the temporal distance, the system applies Kullback-Leibler divergence on the Markov Chain representation to measure the temporal pattern. The two different representations and distances allow the domain experts to decompose and quantify the visual reasoning process temporally and spatially.

Utilizing the stored cases, the system is able to further analyze the eye movement patterns to find significant visual attention sequences for certain groups of people by adapting the method from our previous study by Li et al.^34^. During a visual task, the viewers’ eye movements are naturally segmented by small goals. These segments of subsequences could be repeated in many viewing sessions or by different viewers. With viewer groups defined, the eye movement sequences within a group are binned, segmented, and pooled to find the common and significant subsequences. For one viewer group, the commonly shared subsequences in many cases highlight a necessary visual activity for the task. Such activities can be extracted using frequent pattern mining. With two viewer groups, the system can compare the subsequences in the groups and extract the ones which only appear more frequently in one group using contrast pattern mining^38^. The contrast subsequences are unique to the viewer group and emphasize the differences in visual behavior comparing to the other group.

### Gaze data preprocessing

With the tight correlation between semantic visual information and regions of interest (ROIs), we primarily focus on the sequence of ROIs mapped from the raw gaze data. The fixations are calculated using I-VT^39^ with a $20^{\circ }$ angular speed threshold. Given the complexity of the visual tasks, we also set 100ms as minimum dwell time for fixations.

The proposed system first maps fixation points to the corresponding ROIs defined by domain experts. An example image can be found in the Preprocessing module of Fig. 1 with ROIs marked as yellow polygons with corresponding gaze tracking on top of it in an X-ray image. Given a set of ROI labels $L=\{l_{1},l_{2},l_{3} \cdots \}$, Each fixation point can fall into zero, one or multiple ROIs. Based on the following three rules, the system assigns each fixation to a unique ROI.

- If the fixation falls into an unmarked region, the system will assign this fixation to the ROI with the closest boundary.
- When the fixation is within only one ROI, the system assigns the fixation to this region.
- When the fixation falls into a region of multiple overlapping ROIs, the system assigns the fixation to the ROIs with a minimal area which is likely to be relevant for the reasoning.

With the rules above, the system adds ROI labels to fixations and transfers the eye tracking data from a fixation sequence into a fixation-ROI sequence *S*, which is the basis for visual reasoning quantification and exploration.

Based on the observation that a visual task consists many small subtasks, we further process the fixation-ROI sequences into subsequences for visual reasoning exploration. Let $s_{i} \in L$ be the $i$th fixation in a sequence $S=s_{1}s_{2}\cdots s_{n}$, and $S_{i,j}=s_{i}s_{i+1}\cdots s_{j}$ represent a subsequence of *S* from serial position *i* to *j*. To preserve dwell time information, each label in the sequence is further binned into multiple repeating labels with a 150 ms window. For example, a fixation $s_{i}$ of 445 ms is represented as $s_{i}s_{i}s_{i}$ in the binned sequence. The binned sequence is then segmented into several subsequences of various lengths such as $S_{1,5}$, $S_{1,7}$, $S_{3,8}$, etc. The subsequences are pooled and used for exploring the common and contrast patterns between viewer groups.

### Visual reasoning quantification

With many cases in a library, it is often inefficient to extract patterns for all cases and then go through the important patterns to identify the strategies for certain tasks. Our system provides a quantifiable method to measure the differences between eye movement sequences, thus allowing specialists to quickly identify cases with significant differences between comparison groups. By separating the measurement into temporal and spatial components, it can help specialists determine if the order of ROI visits is important in certain cases.

#### The spatial and temporal representations

After obtaining the corresponding ROI sequence for gaze tracking, the system generates two different representations, spatial and temporal representation, for each eye tracking case. A graph model was created for each case to capture its spatial characteristics. This spatial representation *G* for gaze case is an undirected graph with finite vertices and weighted edges: $G=(V,E)$. In this graph, each vertex represents a ROI in the image and the edges between vertices indicate a direct transition. The weight on each edge measures the linkage between two vertices. Higher weights indicate that the viewers are more likely check the two ROIs together. The first step in spatial representation generation is grouping the fixation points corresponding to the same ROI into a single set. Then, using the ROI sequences, the system adds edges for every direct ROI transition. The weight on an edge equals the total dwell time of the two connected vertices weighted by the number of transitions between them. Finally, the system trims off the self-loop and double edge in the graph to obtain an undirected simple graph.

The temporal representation is a *n*th-order Markov Chain (MC) model with *m* finite states, where *m* is the number of ROIs in one image. Considering the capacity of working memory is limited to about 4 items^40^, we limit the number of *n* to 5. The temporal information is captured by an initial vector $\pi$ and a transition matrix. The initial vector $\pi$ defines the probability of an ROI containing the first fixation. The transition matrix contains the transition probability between two ROIs that will be checked by the viewer consecutively.

To generate the *n*th-order MC model, we assume that the visual attention has the Markov property that the future fixation only depends on the past *n* fixation points. Our temporal representation adapts the MC model to visual reasoning processes by incorporating dwell time into the transition probability to account for the difference in ROI complexity. The transition probability from ROI $l_{i}$ to another ROI $l_{j}$ is calculated as the percentage of transitions to $l_{j}$ out of all transitions from $l_{i}$ weighted by a duration weight of $l_{i}$. The duration weight of $l_{i}$ is the percentage of total dwell time in ROI $l_{i}$ out of all ROIs. Meanwhile, the system determines the initial vector $\pi$ for the MC model by the initial fixation in the corresponding ROI sequence. After obtaining the transition matrix *A* and initial vector $\pi$, the first order MC model is represented in the system. Similarly, the system also constructs higher-order Markov chain models by extending the transition history.

#### The spatial and temporal distances

In this section, we introduce the distances for the two representations. For the spatial representation, we adapt SAGA^37^, a subgraph matching method, to evaluate the spatial similarity. The distance is defined as the weighted sum of three components: subgraph structure ($d_{struct}$), similar nodes ($d_{node}$) and absent nodes ($d_{node\_gaps}$). This spatial similarity is used to produce the spatial results of Table 2.

1 $$\begin{aligned} SD_{\lambda }\left( G_{1},G_{2}\right) =w_{e}d_{struct}+w_{n}d_{node}+w_{g}d_{node\_gaps} \end{aligned}$$

where $w_{s}$, $w_{n}$ and $w_{g}$ are the weights for the three components, and

2 $$\begin{aligned} d_{struct}=\frac{\left| E_{1}-E_{2}\right| }{\left| E_{1}\right| } \qquad d_{node}=\sum _{u\in \hat{V_{1}}}\left( w_{f}d_{att}\left( u,\lambda u\right) +w_{d}d_{dur}\left( u,\lambda u\right) \right) \qquad d_{node\_gaps}=\frac{\sum _{u\in V_{1}-\hat{V_{1}}}gap_{G_{1}}\left( u\right) }{\left| V_{1}\right| } \end{aligned}$$

The structure distance component $d_{struct}$ measures the structural difference between graph representations. It equals the number of unmatched edges from graph $G_{1}$ to another graph $G_{2}$. In node distance $d_{node}$, $\lambda$ is a custom mapping function which matches similar nodes in $G_{1}$ and $G_{2}$ based on the semantic meaning and ontology of the node: $\lambda : {\hat{V}}_{1}\mapsto V_{2}$, where ${\hat{V}}_{1}\subseteq V_{1}$ is a set of matched nodes in $G_{1}$. For example, the individual rib bone nodes are matched to each other rather than to soft tissues. The node distance is calculated based on the fixations distance and duration distance of the nodes:

3 $$\begin{aligned} d_{att}\left( u,\lambda u\right) =\frac{\left| num\,of\,fixation\left( u\right) -num\,of\,fixation\left( \lambda u\right) \right| }{total\_fixation\_num\left( u\right) } \qquad d_{dur}\left( u,\lambda u\right) =\frac{\left| Dwell\,Time\left( u\right) -Dwell\,Time\left( \lambda u\right) \right| }{\sum _{u}Dwell\,Time\left( u\right) } \end{aligned}$$

The node gap $d_{node\_gaps}$ is a penalty for the absent nodes, which can be set by domain experts for each individual node to compensate for semantic significance differences in ROIs. By adjusting the weights of the three components, the spatial distance allows observers to emphasize on the desired visual behavior features.

4 $$\begin{aligned} H\left( \overrightarrow{M_{1}},\overrightarrow{M_{2}}\right) =\sum _{s}\overrightarrow{M_{1}}\left( s\right) \log \frac{\overrightarrow{M_{1}}\left( s\right) }{\overrightarrow{M_{2}}\left( s\right) } \end{aligned}$$

Temporal representation provides time-critical information of gaze activities and produces distance results with high similarity in gaze sequence. When computing temporal distance of two MC representations, their transition matrices *A* are flattened and concatenated with the initial vectors $\pi$ respectively to form $\overrightarrow{M_{1}}$ and $\overrightarrow{M_{2}}$. The system then computes the temporal distance by Kullback-Leibler Divergence as shown in the equation above. The temporal distance is used to capture the temporal characteristics in Table 2. The two distances provide a quantified measurement for eye movement sequences and a quick method for identifying cases with significant temporal or spatial differences between viewer groups.

### Visual reasoning pattern extraction

Due to uncontrollable micro-saccades or task-unrelated fixation on salient regions, the visual attention sequence from subjects can be noisy. It is unrealistic to manually go through the eye tracking recordings in each case to filter out noise and segment the task related subsequences. In some cases, the important fixation sequences are subconscious, which makes it even less feasible for conscious manual processing. With accumulated cases in the library, the system can find repeated segments of subsequences that are commonly shared among a certain group of predefined viewers. A high frequency highlights the potential significance of the subsequences for further examination by domain experts. The system adapts the method of frequent pattern mining and contrast pattern mining^38^ to eye movement sequences to produce these high potential subsequences. The results of subsequence of ROI fixations are readable and explainable to enhance existing knowledge of visual tasks.

After preprocessing, the ROI sequences are binned and segmented into subsequences, and the system pools them together according to the user-defined viewer groups. To compensate for the small variations in the subsequences that are caused by micro-saccades or random stray fixations, our method allows a small amount of differences in the subsequences. We use Levenshtein distance when calculating the distance between two sequences $d_{lev}(S_{i,j},S_{x,y})=lev_{S_{i,j},S_{x,y}}(|S_{i,j}|,|S_{x,y}|)$. When mining the common and contrast visual reasoning patterns, two subsequences are considered similar if their distance is smaller than a predefined threshold $\theta$, which can be adjusted according to different applications.

The common visual reasoning patterns are defined as the subsequences that are commonly shared by two groups of viewers, which means that the subsequences exist in a certain percent of cases in both groups. This percentage that the set of cases of one viewer group ${\mathbb{S}}={S^{1},S^{2},\ldots ,S^{N}}$ have a subsequence $S_{i,j}$ is defined as the support value of the subsequence. The support value is used to calculate the average frequency in Table 3.

5 $$\begin{aligned} supp\left( S_{i,j},{\mathbb{S}} \right) =\frac{\left| \left\{ S^{k}: d_{lev}(S_{i,j},S_{x,y}) \le \theta , S_{x,y}\in S^{k} \right\} \right| }{N} \end{aligned}$$

If a similar subsequence exists in a case with no exact matches, it is still considered present for the subsequence when calculating its support. However, if the occurrence of the subsequence is drastically different in two groups, it is more significant for the group with much higher support. To ensure a subsequence is common in both groups, the support values in the two groups must be comparable. The ratio of the two support values of $S_{i,j}$ in the two groups is defined as growth. The growth value is used to determine the percentage of unique patterns in Table 3.

6 $$\begin{aligned} grow\left( S_{i,j},{\mathbb{S}}^{1}\right) = \left\{ \begin{matrix} \min _{S_{x,y}}\left( \frac{supp(S_{i,j},{\mathbb{S}}^{1})}{supp(S_{x,y},{\mathbb{S}}^{2})} \right) , &{} \exists S_{x,y} \in {\mathbb{S}}^{2}, d_{lev}(S_{i,j},S_{x,y}) \le \theta \\ \texttt {inf}, &{} \forall S_{x,y} \in {\mathbb{S}}^{2}, d_{lev}(S_{i,j},S_{x,y}) > \theta \end{matrix}\right. \end{aligned}$$

The growth is calculated with the similar subsequence with the maximum support value, which means the growth of a subsequence takes the minimum value calculated with all the similar matches. To qualify as a common visual reasoning pattern, a subsequence’s supports needs to pass the support threshold $\rho$ for both groups while keeping its growth below the growth threshold $\epsilon$. The common patterns for both groups is ${\mathbb{C}}_{common}=\{S_{i,j}\in {\mathbb{S}}^{1} \cup {\mathbb{S}}^{2} | supp(S_{i,j},{\mathbb{S}}^{1})\ge \rho , supp(S_{i,j},{\mathbb{S}}^{2})\ge \rho , grow(S_{i,j},{\mathbb{S}}^{1})< \epsilon , grow(S_{i,j},{\mathbb{S}}^{2})< \epsilon \}$.

A contrast visual reasoning pattern, however, is a subsequence which occurs much more commonly in only one viewer group. According to the definition, the growth of the subsequence needs to exceed the growth threshold for the group, and the support for that group must be greater than the support threshold. The contrast patterns for ${\mathbb{S}}^{1}$ is ${\mathbb{C}}_{contrast}=\{S_{i,j}\in {\mathbb{S}^{1}} | supp(S_{i,j},{\mathbb{S}^{1}})\ge \rho , grow(S_{i,j},{\mathbb{S}^{1}})\ge \epsilon \}$.

In addition to the Levenshtein distance, we also measure the similarity between patterns based purely on visited ROIs using bag-of-words representation and cosine distance. The bag-of-words vector of pattern $S_{i,j}$ is denoted as $B_{i,j}=[b_{1},b_{2},b_{3} \cdots ]$, where $b_{p}=|\{ s_{q}=l_{p} : s_{q}\in S_{i,j}, l_{p}\in L \}|$. The ROI distance is $d_{roi}\left( S_{i,j},S_{x,y}\right) =\left( 1-B_{i,j}B_{x,y}/\Vert B_{i,j}\Vert _{2}\Vert B_{x,y}\Vert _{2} \right)$. This ROI distance is not used to extract patterns but to assess them in post-generation analysis.

The common visual reasoning patterns reveal the commonalities in visual behaviors between two viewer groups, and the contrast visual reasoning patterns highlight the unique eye movements for each group. The combination of these two types of patterns provides insights into the reasoning processes and strategies for solving complex visual tasks.

## Results

As explained the Method section, the system should help researchers effectively identify spatial and temporal differences in visual reasoning processes and provide significant common and contrast patterns for further examination. To test for effectiveness, an experiment was conducted to compare visual reasoning with subjects at varying expertise levels.

### Experiment configurations

Informed consent was obtained from 39 subjects from two major viewer groups including 15 registered radiographers (experts, more than 10,000 h of clinical experience) and 24 students (novices) in the Radiography department at the University of Missouri. The novice group was recruited from the same bachelor-level program and further divided into 11 seniors (completed all image analysis coursework and over 1000 h of experience) and 13 juniors (less than 400 h, training in progress). The results are produced by comparing the eye movement recordings between the groups. An EyeTribe eye tracker^41^ with 60 Hz sampling rate was applied to collect the viewers’ eye movements. The questions and images were shown on a 27-inch monitor placed about 60 cm away from participants.

The experiment included 10 X-ray images with 10 questions. All images used in the study were obtained from the teaching files maintained by the Radiography department and had been previously de-identified to ensure patient privacy. The images used in this experiment contained no personally identifiable patient information and their use does not violate HIPAA regulations. Each image was chosen for a question in the modified 10-level visual structure^42^ (Table 1) which groups visual tasks from low syntactic levels to high semantic levels. The progression of difficulty imitates the normal reasoning process of a radiographer approaching a new image. The participants viewed the task question prior to initiating the 15 seconds of image viewing and eye movements recording. They were then asked to explain their answers and reasons which are recorded and transcribed. The experiment was approved by the University of Missouri Health Science Institutional Review Board (IRB #2001653), and all methods were performed in accordance with the relevant guidelines and regulations. The fixation statistics can be found in Supplementary document.

**Table 1:** 10-Level knowledge structure with corresponding experiment questions.

Task	Visual Knowledge Level	Questions
1	Type Technique	What is the modality of this image?
2	Global Distribution	Describe the overall photographic properties of this image.
3	Local Structure	What basic textual elements do you identify on this image?
4	Global Composition	How do you orient yourself to this image?
5	Generic Objects	What body part does this image demonstrate?
6	Generic Scene	What is the projection of this image?
7	Specific Objects	Identify 3 foreign objects on this image
8	Specific Scene	Evaluate the positioning of this image.
9	Abstract Objects	Describe this patient based on what you see in this image
10	Abstract Scene	What problem(s) do you think this patient has?

### Assessing spatial and temporal characteristics

We tested our distance model on all viewer groups. Our hypothesis is that for a specific distance, there should be a high similarity with intragroup visual reasoning processes and a large difference for intergroup visual reasoning processes. We performed the following group comparisons: Expert vs. Expert (EE), Expert vs. Senior (ES), Expert vs. Junior (EJ) and Expert vs. Novice (EN). These comparisons were performed using all-against-all distance measurement between and within groups. The Expert vs. Expert (EE) distances allow us to establish the level of internal consistency within the expert group. The Mann-Whitney U test was used on combinations of these four group comparisons because it has no requirement on data distribution. A p-value less than 0.05 indicates that two distance distributions are statistically different. More details about the distances are shown in Supplementary document.

The statistical test results are shown in Table 2 and the significant cases are highlighted. For spatial distances, we give equal weight to each component. We can see that experts’ visual reasoning patterns are different from those of novices in all cases based on spatial distance. In the junior and senior groups, we can see that there are significant differences between junior and senior students’ gaze patterns compared to experts’ patterns at low-level tasks (Tasks 1–3). But for most high-level tasks (Tasks 4–10), senior and junior students perform similarly, which demonstrates the experience gap between novices and experts. Similar to the spatial distance testing, the Mann-Whitney U test was applied to the temporal distances. Compared to the spatial component results, we have similar observations from the experiments using the temporal component. For the novice group, junior students who have one less year of training are close in performance to the senior students for most of the tasks. This could be explained as the visiting order of ROIs may not be important for both novice student groups for image understanding. On the other hand, experts perform differently comparing to both junior and senior students. With well-trained knowledge, experts can quickly locate relevant regions in the images and perform quite differently from novice students in temporal visual activities.

**Table 2:** *p*-value for Mann–Whitney test on all group comparisons with overall spatial distance and Kullback–Leibler temporal distance.

Task	Spatial Distance				Temporal Distance			
	EE/EN	EE/EJ	EE/ES	EJ/ES	EE/EN	EE/EJ	EE/ES	EJ/ES
1	<** 0.001**	<** 0.001**	**0.003**	< **0.001**	< **0.001**	<** 0.001**	**0.008**	**0.036**
2	<** 0.001**	<** 0.001**	< **0.001**	0.693	**0.003**	**0.011**	**0.004**	0.703
3	<** 0.001**	<** 0.001**	**0.006**	**0.019**	<** 0.001**	<** 0.001**	< **0.001**	**0.001**
4	<** 0.001**	<** 0.001**	0.228	<** 0.001**	<** 0.001**	<** 0.001**	< **0.001**	**0.017**
5	**0.034**	0.074	**0.040**	0.699	<** 0.001**	<** 0.001**	**0.003**	**0.012**
6	<** 0.001**	<** 0.001**	<** 0.001**	0.656	<** 0.001**	<** 0.001**	< **0.001**	0.365
7	<** 0.001**	<** 0.001**	<** 0.001**	0.097	<** 0.001**	<** 0.001**	<** 0.001**	**0.009**
8	<** 0.001**	**0.004**	<** 0.001**	0.498	<** 0.001**	<** 0.001**	<** 0.001**	0.502
9	<** 0.001**	<** 0.001**	<** 0.001**	<** 0.001**	**0.003**	**0.001**	**0.049**	0.143
10	**0.011**	**0.019**	**0.020**	0.729	**0.021**	0.056	**0.020**	0.553

Bold numbers highlight a *p*-value smaller than 0.05.

### Explaining visual reasoning patterns

With the significant cases highlighted by the two distance measurements, we explored the common and contrast visual reasoning patterns to investigate how the expertise groups differ in different tasks. We analyzed eye movement sequences with the following comparisons: Expert vs. Novice (EN), Expert vs. Senior (ES), Expert vs. Junior (EJ), and Senior vs. Junior (SJ). We set the minimal length of a subsequence to 4 ROIs (400 ms). To compensate for small variations caused by micro-saccade or stray fixations, we consider two subsequences similar if their Levenshtein distance is no greater than 2. The support threshold is set at 0.2, and the growth threshold is set to 2.

The eye movement subsequences identified can be characterized by the number of contrast patterns, average and maximum pattern lengths, average pair-wise Levenshtein and ROI distances, average support value, and percentage of pattern with infinite growth of EN comparison, as shown in Table 3. The number of patterns and pattern lengths vary depending on the task. Some tasks can be finished by checking different ROIs of the same clinical significance, which could result in fewer patterns. Most patterns are unique in one group with an infinity growth rate. The patterns also exhibit an average length of 7–9, indicating the capability of capturing meaningful visual reasoning patterns which can characterize an expertise group. Task 8 stands out with the fewest patterns and shortest lengths, indicating that the visual reasoning processes are highly diverse even within a group. The longer average pattern lengths for experts in most tasks reflect higher unity in their sequences. Also, the phenomenon in which experts’ patterns generally have smaller ROI distances than novices’ shows that the experts are more efficient by focusing on less ROIs. The larger Levenshtein distances for experts indicate that they develop different orders of ROI examination in practice which may not conform with textbook instructions followed by novices. The patterns, along with the think-aloud recordings, are examined and interpreted by an experienced radiographer to identify meaningful differences in visual reasoning behaviors.

**Table 3:** The number of contrast patterns ($\#seq$), average and maximum pattern lengths (${\overline{l}}$, $l_{max}$), average pair-wise Levenshtein and ROI distance between patterns within a group ($d_{lev}$, $d_{roi}$), average support value (${\overline{supp}}$), and the percentage of patterns with infinity growth ($\%uniq$, unique subsequences for the group) of extracted contrast visual reasoning patterns for Expert and Novice groups in Expert vs. Novice comparison.

Task	Expert|ExpertNovice							Novice|ExpertNovice						
	$\#seq$	${\overline{l}}$	$l_{max}$	$d_{lev}$	$d_{roi}$	${\overline{supp}}$	$\%uniq$	$\#seq$	${\overline{l}}$	$l_{max}$	$d_{lev}$	$d_{roi}$	${\overline{supp}}$	$\%uniq$
1	19	8.42	16	8.532	0.604	0.407	68.4%	28	7.86	16	7.780	0.636	0.357	75.0%
2	101	7.59	16	7.671	0.769	0.272	76.2%	41	6.20	13	6.690	0.806	0.296	92.7%
3	115	8.64	16	7.955	0.536	0.275	53.9%	71	7.00	10	6.601	0.644	0.326	71.8%
4	28	8.07	13	7.722	0.722	0.281	46.4%	47	8.98	13	6.669	0.313	0.318	63.8%
5	41	7.95	16	7.706	0.589	0.289	53.7%	36	6.92	13	7.148	0.671	0.326	75.0%
6	50	8.26	16	8.607	0.755	0.269	42.0%	54	6.33	13	6.628	0.776	0.322	53.7%
7	97	9.41	19	8.619	0.565	0.256	43.3%	34	6.38	13	6.783	0.793	0.321	73.5%
8	9	4.33	7	4.528	0.912	0.252	77.8%	28	4.54	7	4.667	0.836	0.318	71.4%
9	35	7.26	13	8.525	0.616	0.338	57.1%	65	5.85	10	6.974	0.682	0.362	70.8%
10	65	9.17	16	6.793	0.627	0.288	38.5%	37	7.24	13	6.259	0.796	0.295	62.2%

**Figure 2:**
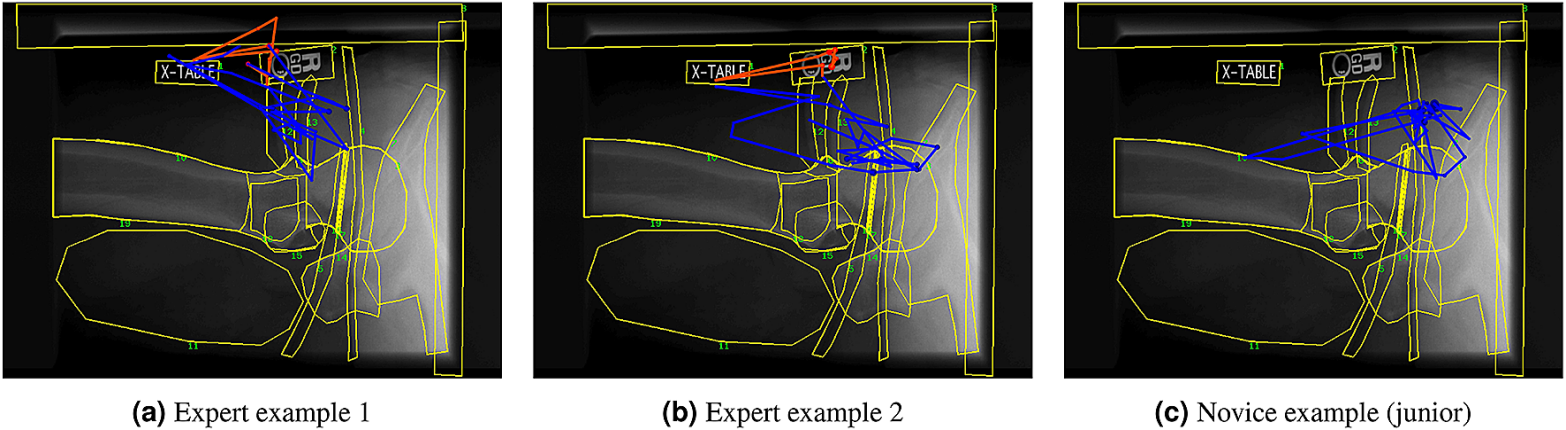
Example eye movement sequences for Task 4. ROIs are marked with yellow lines. The connected blue lines and circles on each image show an entire eye movement sequence by one participant. Each circle is a fixation in the sequence, and its size represents the fixation duration. The red subsequences highlight the contrast pattern by experts which checks the R marks and the label. The sequences are plotted with Matplotlib^43^.

Not only can the subsequence patterns reveal the visual reasoning common to an expertise group, they can also reveal details regarding the visual tasks undertaken. Tasks 1 to 3 only require fundamental radiology knowledge and concern basic image features like contrast and exposure, so the viewers might fixate on any region regardless of medical significance for the same visual information. At the medium levels, the contrast patterns and common patterns start to show correlations with expertise. Task 4 asks the viewer about the projection orientation while showing a picture of the right femoral head and pelvis (Fig. 2). Unlike the low-level tasks, this task requires a moderate amount of radiology and medical knowledge, and the viewer needs to visit certain ROIs. Additionally, the image is projected from the side of the pelvis, which obscures the structure of the femoral head and makes it less distinguishable than it is in other projections. This less common projection exposes the visual reasoning differences between expertise groups. All groups exhibit short common patterns (around 1350 ms) of examining the orientation of the femoral head with pelvis, but the average support is generally lower in experts than in novices (0.33 vs. 0.47). Longer patterns (above 2000 ms) only show up in novices’ sequences as contrast patterns. The prolonged fixations exhibited by the novices, especially juniors, reveal their lack of experience, and that additional examination is required. Most novices commented that they did not see anything abnormal. The experts exhibit several unique contrast patterns with extended fixations on the “R” marker (noted in red in Fig. 2a,b). The three dots at the bottom of the circle indicate that the image is being taken with a cross-table projection where the image receptor is standing up perpendicularly to the patient table. Seniors also show some contrast patterns that indicate learning progression when comparing the patterns against juniors. They have longer fixations on the greater and lesser trochanter, which tell seniors that the image is in a lateral projection with the x-ray beam traveling from the patient’s inner thigh through his outer thigh. The experts share some shorter fixations of trochanters with the seniors, but the patterns generally contain fixations on the “R” marker to confirm the two pieces of orientation information.

**Figure 3:**
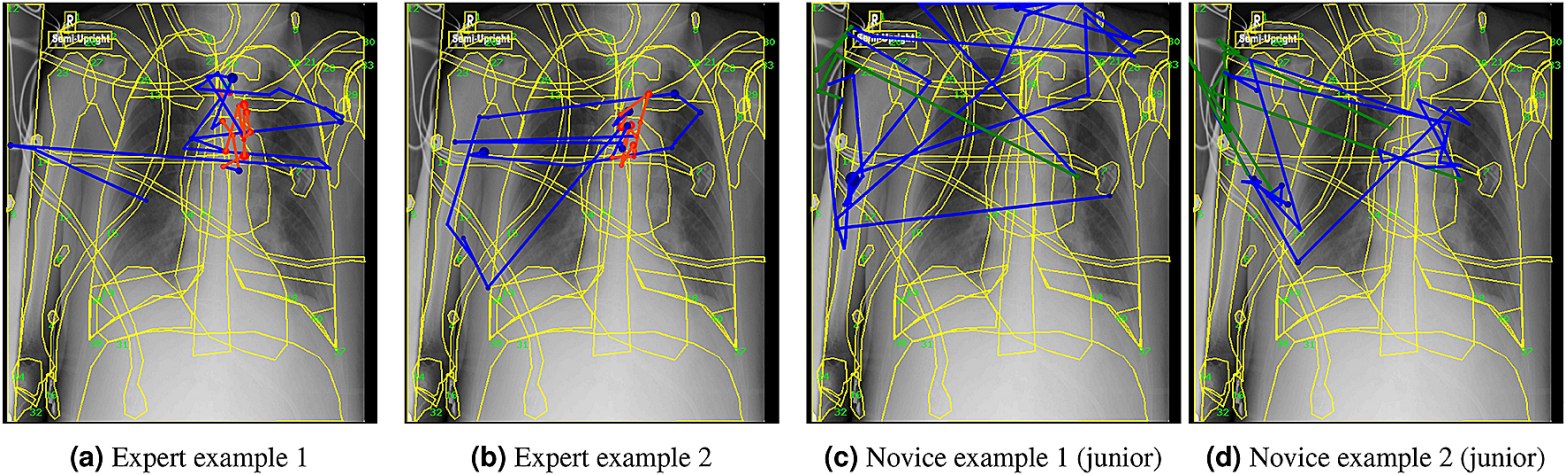
Example eye movement sequences for Task 7. The red subsequences of experts show close examination of the potential diseased area, while the novices are distracted by the technical imperfections at the left edge (green subsequences).

As task complexity increases, the differences between expertise groups become more evident. The chest x-ray presented with Task 7, “Identify 3 foreign objects”, has complex features and objects (Fig. 3), and it exposes more interesting commonalities and differences. The tubes and clips across the patient’s chest are obvious foreign objects, and they appear in all expertise groups as common patterns with support values above 0.5. The juniors pay the most attention to the right humerus, where there are multiple wires and snaps (Fig. 3c). One contrast pattern shared only among the novices is the prolonged fixation (above 1050 ms) on the collimated edge where the x-ray exposure ends (Fig. 3c,d). The wedge-shaped line and shadow extending from the upper left corner of the image were created by a misaligned x-ray tube and image receptor. It indicates that this image was performed in the patient’s room. Such a situation rarely occurs in a dedicated x-ray room where most x-rays are performed. These misaligned images are novel to the novices, so the collimated edge attracts their visual attention, which is confirmed in their comments. However, the experts have become accustomed to this occurrence and do not focus on it. With extended experience and medical knowledge, the experts show contrast patterns which dwell on the hilum and the left lung (Fig. 3a,b). The cloudiness in the hilum and left lung area suggests either pneumonia or atelectasis. The image feature requires familiarity with chest anatomy so the pathology can be detected, and the contrast pattern of the expert exposes the key differences.

**Figure 4:**
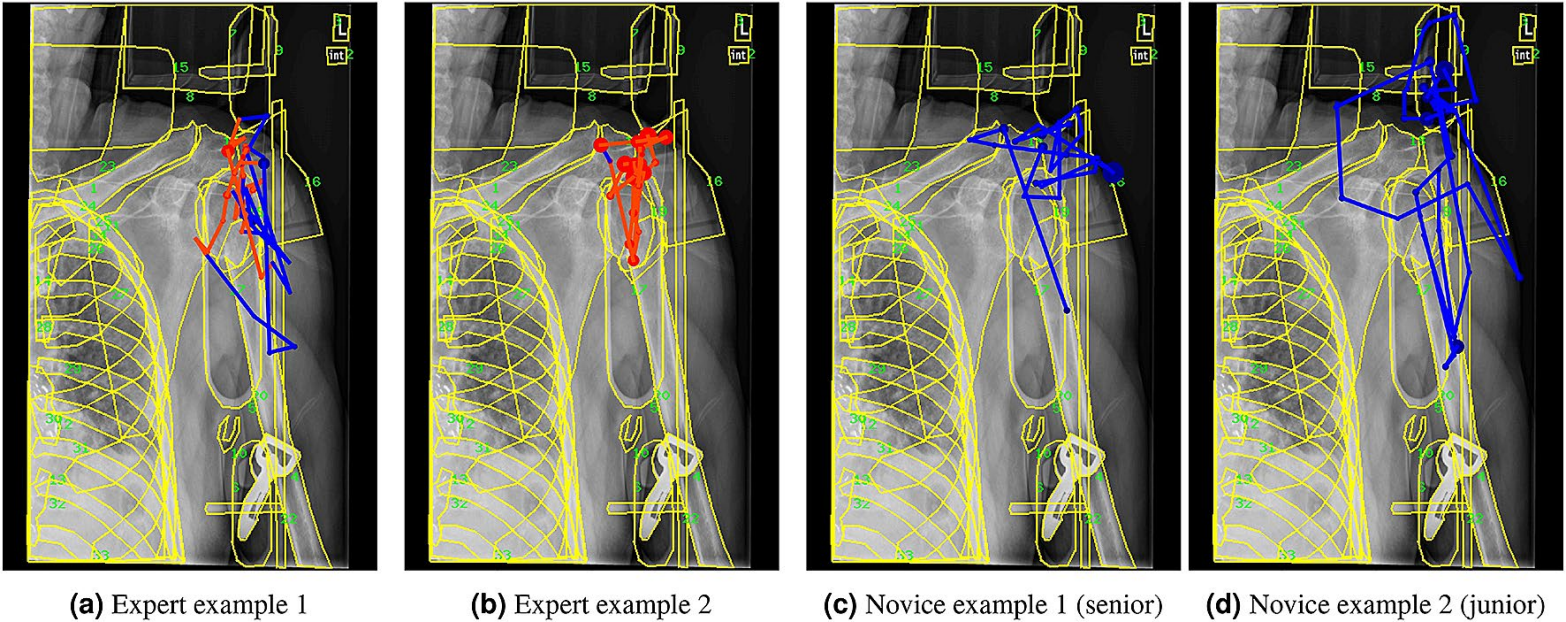
Example eye movement sequences for Task 10. Unlike novices, the experts are not distracted by the non-anatomical structures and closely examine the fracture of the humeral head as shown by the red subsequences.

Increased task complexity also requires increases in dwell times and the ability to prioritize visual focus on relevant ROIs. Task 10 requires a high level of anatomy and radiology knowledge to answer, and these knowledge differences are evident in the patterns of each expertise group. When compared to novices, the experts have unique contrast patterns with much longer fixation sequences on the humeral head, humerus, and the area where humerus was impacted into humeral head (Fig. 4a,b). The extended dwell time (above 2400 ms) and constant cross examination indicates that they were examining the compression fracture of the humeral head, which is confirmed by mentioning traumatic injury by all experts. The common patterns novices share with experts show that they frequently look at the humerus and humeral head as well, although the average support is much lower (0.38 vs. 0.55). The compression fracture is not easily recognized and thus skimmed through by the novices because the outline of the bone remains basically normal. It is fine textural changes that the experts were targeting. However, the seniors show a higher support value of these shorter common patterns than juniors (0.55 vs. 0.44), suggesting that more seniors are starting to check critical areas. This is supported by some seniors’ comments on the trauma. The experience progression is also highlighted by some seniors’ unique patterns focused on an immobilizing sponge (Fig. 4c). The sponge is very similar in brightness and contrast to the surrounding tissues, making it less noticeable than high-contrast non-anatomical structures like the clip of the safety strap. The seniors have learned to ignore obvious distractions, but their attention is still captured by less familiar, but still irrelevant, objects. The contrast patterns for juniors, most of who commented mainly on unrecognized artifacts, show the greatest amount of distraction by non-anatomical structures such as backboard edge and handle (Fig. 4d). These non-anatomical structures are unusual and thus salient to novices, but they are limited in assisting with the task. For experts, their expertise is revealed by the high concentration of task-effective fixations in their contrast patterns.

The extracted common and contrast patterns not only explain the spatial and temporal differences in visual reasoning processes, they also unveil subtle experience progression that can only be found in segments of the whole eye movement sequences. The visual reasoning explanations of the three cases are based on a portion of the extracted patterns in Table 3. The extracted patterns for other high-level tasks also demonstrate different reasoning processes between expertise groups, illustrating the potential for applying the method to more cases, as well as to other medical image areas, for a systematic and comprehensive understanding of human visual reasoning.

## Discussion

This paper reports a computational method that may bring the research community one step closer to designing explainable computational tools that can explain human visual reasoning. Previous studies have shown that experts perceive diagnostic-related regions more quickly^44^ and allocate their attention more efficiently^45^. Using the results from our method, we can go one step further and carefully examine significant segments of eye movement and the underlying anatomical meaning when comparing different tasks and groups of viewers. This method is especially powerful in cases where some visual reasoning processes are subconscious and cannot be fully explained. Such processes are prone to be overlooked when presented in a prolonged series of eye movements. Our system quantitatively characterizes visual reasoning processes to quickly highlight important cases, and the extracted common and contrast patterns can lead to new knowledge discovery.

Examining the spatial and temporal distance comparison results and extracted common and contrast patterns, we found that experts and novices tend to use different visual patterns to solve the same high-level tasks. In the novice group, we observed that junior and senior students have minimal significant differences when dealing with most mid- to high-level tasks based on the entire eye movement sequences. However, the contrast patterns show some small differences in subsequences when comparing the two novice groups, which indicate some progression in experience with seniors. This small difference is often overshadowed by the overall characteristics. It is only when the common and contrast patterns are analyzed that we are able to detect the detailed differences in visual reasoning processes between the groups.

In general, the spatial and temporal representations and distances allow us to quantitatively determine the statistical difference in visual behaviors. The extracted common and contrast patterns provide readable and explainable subsequences and enable the domain experts to investigate the visual strategies in detail. This retrospective study shows promise for discovering implicit visual strategies gained through experience and may lead to new, evidence-based knowledge. By capturing and explaining the steps involved in visual reasoning for specific tasks, we can better support those who engage regularly in complex visual reasoning. In the educational setting, it is valuable for formulating more efficient instruction, highlighting avoidable distractions, and improving professional practices. In healthcare delivery, it holds promise for reducing diagnostic discrepancies and improving health outcomes.

We believe that our attempt to use computational means to quantify and understand the human visual reasoning process may provide the community with a starting point to systematically capture the implicit visual reasoning processes. The capability of a system with explainable results would bring potentially valuable and novel insights in visual strategies to fields that rely on image-based diagnosis such as dermatology, pathology and radiology. It will also allow us to gain potential direction for designing explainable artificial intelligence algorithms based on accumulated human knowledge that is supported by the evidence in the recorded visual attention of experts. In the future, the distances can be utilized to enhance content-based medical image retrieval systems^46^ to search and retrieve similar visual reasoning sequences given a case or an image. The system can also be adapted for other visually-intensive tasks such as geospatial information retrieval^47^.

## Supplementary information

Supplementary Information.
