# Supplementary material for: Computational modeling of human reasoning processes for interpretable visual knowledge: a case study with radiographers
Source: Sci Rep. 2020 Dec 10;10:21620. doi: 10.1038/s41598-020-77550-9 (PMC7730148; doi:10.1038/s41598-020-77550-9)
Supplement: Supplementary file 1 — Supplementary Information. [file 41598_2020_77550_MOESM1_ESM.pdf]

Computational Modeling of Human Reasoning Processes for Interpretable Visual Knowledge – A Case Study with Radiographers

Yu Li[1], Hongfei Cao[1], Carla M. Allen[3], Xin Wang[4], Sanda Erdelez[5], and Chi-Ren Shyu[1,2,*]

[1]Department of Electrical Engineering and Computer Science, University of Missouri, Columbia, Missouri, 65211, USA
[2]Institute for Data Science and Informatics, University of Missouri, Columbia, Missouri, 65211, USA
[3]Department of Clinical and Diagnostic Science, University of Missouri, Columbia, Missouri, 65211, USA
[4]Department of Information Science, University of Northern Texas, Denton, Texas, 76203, USA
[5]School of Library and Information Science, Simmons University, Boston, Massachusetts, 02115, USA
[*]shyuc@missouri.edu

Supplementary Material

	Expert		Novice		Junior		Senior	
Task	num	$\overline{dur}$	num	$\overline{dur}$	num	$\overline{dur}$	num	$\overline{dur}$
1	408	275.578	637	284.080	377	272.506	260	300.862
2	665	265.086	929	294.672	470	318.927	459	269.836
3	1451	270.681	2503	258.422	1487	258.352	1016	258.525
4	561	266.527	1061	259.938	610	255.547	451	265.877
5	671	304.960	1001	331.805	565	337.343	436	324.630
6	828	313.480	1430	335.061	770	341.443	660	327.614
7	942	315.213	1336	294.834	692	301.830	644	287.317
8	608	236.801	1090	236.004	627	242.003	463	227.879
9	749	286.162	1396	252.784	814	253.184	582	252.226
10	868	316.055	1410	305.343	770	310.133	640	299.579

Table S1. Number of fixations (num) and average fixation duration ($\overline{dur}$, ms) for each expertise group in different tasks.

	EE		EN		ES		EJ		SJ	
Task	μ	SE	μ	SE	μ	SE	μ	SE	μ	SE
1	13.445	0.228	15.334	0.133	14.475	0.197	15.860	0.172	17.195	0.200
2	16.888	0.223	18.005	0.139	18.013	0.205	17.995	0.170	18.966	0.219
3	16.046	0.183	16.939	0.088	16.799	0.125	17.080	0.123	17.652	0.120
4	15.791	0.190	16.703	0.095	16.002	0.132	17.337	0.132	17.506	0.140
5	15.335	0.192	15.786	0.089	15.834	0.127	15.729	0.125	16.017	0.114
6	14.710	0.119	16.124	0.083	16.103	0.109	16.148	0.128	17.728	0.151
7	16.010	0.222	17.012	0.096	16.855	0.128	17.184	0.144	18.146	0.104
8	19.480	0.262	20.494	0.116	20.531	0.161	20.455	0.167	20.766	0.136
9	17.860	0.259	19.634	0.127	19.124	0.182	19.995	0.171	20.824	0.163
10	12.915	0.170	13.513	0.093	13.636	0.143	13.391	0.118	14.211	0.137

Table S2. Mean (μ) and standard error (SE) of the spatial distances used for Mann-Whitney U test.

	EE		EN		ES		EJ		SJ	
Task	μ	SE	μ	SE	μ	SE	μ	SE	μ	SE
1	1.131	0.159	1.923	0.084	1.682	0.129	2.070	0.111	2.507	0.122
2	2.432	0.191	2.893	0.104	2.936	0.140	2.832	0.156	2.398	0.134
3	2.454	0.138	3.316	0.063	3.058	0.079	3.574	0.098	3.280	0.084
4	2.556	0.141	3.199	0.067	2.995	0.091	3.383	0.098	2.892	0.079
5	2.038	0.108	2.603	0.061	2.509	0.089	2.713	0.082	2.574	0.085
6	1.393	0.078	2.420	0.066	2.365	0.081	2.485	0.107	2.834	0.094
7	1.601	0.098	2.590	0.067	2.412	0.087	2.786	0.102	2.630	0.077
8	1.677	0.124	2.720	0.064	2.621	0.082	2.824	0.100	3.528	0.093
9	1.900	0.143	2.456	0.081	2.273	0.111	2.585	0.113	2.379	0.099
10	1.824	0.139	1.930	0.055	2.005	0.081	1.855	0.073	1.998	0.075

Table S3. Mean (μ) and standard error (SE) of the temporal distances used for Mann-Whitney U test.

Task	Expert	Novice	Junior	Senior
1	1521	2593	1415	1178
2	5294	8336	4969	3367
3	16728	29209	18492	10717
4	1966	4365	2533	1832
5	3994	6631	4047	2584
6	6936	14472	8004	6468
7	8868	9472	4857	4615
8	1862	3638	2316	1322
9	6766	11066	6404	4662
10	7571	11566	6552	5014

Table S4. The number of processed subsequences before common and contrast visual reasoning extraction.
